# Ameliorating the Developmental Neurotoxicity of Chlorpyrifos: A Mechanisms-Based Approach in PC12 Cells

**DOI:** 10.1289/ehp.10194

**Published:** 2007-06-14

**Authors:** Theodore A. Slotkin, Emiko A. MacKillop, Ian T. Ryde, Frederic J. Seidler

**Affiliations:** Department of Pharmacology & Cancer Biology, Duke University Medical Center, Durham, North Carolina, USA

**Keywords:** adenylyl cyclase, brain development, chlorpyrifos, cholinergic antagonists, neurotoxicity, nicotine, organophosphate insecticides, oxidative stress, theophylline, vitamin E

## Abstract

**Background:**

Organophosphate developmental neurotoxicity involves multiple mechanisms converging on neural cell replication and differentiation.

**Objectives:**

We evaluated mechanisms contributing to the adverse effects of chlorpyrifos (CPF) on DNA synthesis, cell number and size, and cell signaling mediated by adenylyl cyclase (AC) in PC12 cells, a neuronotypic cell line that recapitulates the essential features of developing mammalian neurons.

**Results:**

In undifferentiated cells, cholinergic receptor antagonists had little or no protective effect against the antimitotic actions of CPF; however, when nerve growth factor was used to evoke differentiation, the antagonists showed partial protection against deficits in cell loss and alteration in cell size elicited by CPF, but were ineffective in preventing the deterioration of AC signaling. Nicotine, which stimulates nicotinic acetylcholine receptors but also possesses a mixture of prooxidant/antioxidant activity, had adverse effects by itself but also protected undifferentiated cells from the actions of CPF and had mixed additive/protective effects on cell number in differentiating cells. The antioxidant vitamin E also protected both undifferentiated and differentiating cells from many of the adverse effects of CPF but worsened the impact on AC signaling. Theophylline, which prevents the breakdown of cyclic AMP, was the only agent that restored AC signaling to normal or supranormal levels but did so at further cost to cell replication.

**Conclusions:**

Our results show definitive contributions of cholinergic hyperstimulation, oxidative stress, and interference with AC signaling in the developmental neurotoxicity of CPF and point to the potential use of this information to design treatments to ameliorate these adverse effects.

The nearly ubiquitous exposure of the human population to organophosphate pesticides has raised increasing concern about their propensity to elicit developmental neurotoxicity at exposures that go undetected because of the absence of systemic signs of intoxication (Colborn 2006; Costa 2006; Landrigan 2001; Mileson et al. 1998; Slotkin 2005; Weiss et al. 2004). At high doses, inhibition of cholinesterase leads to cholinergic hyperstimulation and its associated symptoms (Mileson et al. 1998), but the developing brain is targeted by these agents at much lower exposures. In large measure, the vulnerability of the fetus and neonate reflects interference with basic processes of neural cell replication and differentiation, culminating in aberrant synaptic function and associated behavioral deficits (Barone et al. 2000; Pope 1999; Rice and Barone 2000; Slotkin 2004). Importantly, many of these effects involve mechanisms unrelated to cholinesterase inhibition and are elicited even at exposures below the threshold for anticholinesterase actions (Pope 1999; Slotkin 2004, 2005). In fact, rather than operating through a single defined mechanism, organophosphates such as chlorpyrifos (CPF) actually disrupt brain development through several families of mechanisms, including direct interactions with acetylcholine receptors, interference with intracellular signaling cascades that transduce receptor signals on the cell surface into intracellular events, and oxidative stress (Gupta 2004; Slotkin 1999, 2004, 2005; Yanai et al. 2002).

The diversity of mechanisms underlying the developmental neurotoxicity of CPF and the organophosphates is one of the main reasons for the wide developmental window over which the immature brain remains vulnerable to these agents, ranging from the earliest stages of brain formation through the postnatal consolidation of synaptic connections (Pope 1999; Slotkin 2004, 2005). For the same reasons, then, the standard countermeasures against acute organophosphate poisoning, which are aimed at the immediate reactivation of cholinesterase and prevention of seizures (Shih et al. 2003; Shih and McDonough 1999), are unlikely to prove effective in preventing the adverse effects on brain development associated with lower exposures of the population at large. In the present study, we assessed the relative contributions of four distinct mechanisms in the developmental neurotoxicity of organophosphates by examining the ability of targeted therapies to ameliorate the effects of CPF: the combination of atropine plus mecamylamine (antagonists toward muscarinic and nicotinic acetylcholine receptors, respectively); nicotine, which both stimulates and desensitizes nicotinic receptors but also has prooxidant and antioxidant characteristics (Gitto et al. 2002; Guan et al. 2003; Qiao et al. 2005; Xie et al. 2005) and promotes the release of neurotrophic factors (Belluardo et al. 2000); the antioxidant vitamin E; and theophylline, a phosphodiesterase inhibitor that prevents the breakdown of cyclic AMP. Each of these has a specific rationale for potential use for amelioration of organophosphate-induced developmental neurotoxicity. Inhibition of cholinesterase produces cholinergic hyperstimulation because the excess acetylcholine interacts with muscarinic and nicotinic receptors, and the organophosphates themselves interact directly with the receptors (Gupta 2004). In addition to its direct actions (stimulatory and inhibitory) on nicotinic receptors, nicotine can protect neural cells by blunting the antimitotic and prooxidant effects of CPF (Qiao et al. 2003, 2005). Because CPF produces part of its neurotoxicity through oxidative mechanisms (Bagchi et al. 1995; Crumpton et al. 2000b; Gupta 2004; Jett and Navoa 2000; Qiao et al. 2005; Slotkin et al. 2005), classical antioxidants such as vitamin E may also be protective, just as they are against other oxidative stressors (Gultekin et al. 2001; Qiao et al. 2005). Finally, interference with the G-protein/adenylyl cyclase (AC) signaling cascade, which is responsible for the synthesis of cyclic AMP, is one of the main noncholinergic pathways by which CPF evokes damage to the developing brain (Slotkin 1999, 2004, 2005; Yanai et al. 2002). Accordingly, we hypothesized that theophylline might have ameliorating effects against the actions of CPF.

We conducted our evaluations in PC12 cells, a standard neurodevelopmental model (Teng and Greene 1994) that reproduces many of the key mechanisms and features of the adverse effects of CPF on the developing brain *in vivo* (Bagchi et al. 1995, 1996; Crumpton et al. 2000a, 2000b; Das and Barone 1999; Flaskos et al. 1994; Jameson et al. 2006b; Li and Casida 1998; Nagata et al. 1997; Qiao et al. 2001, 2005; Slotkin 1999, 2004, 2005; Song et al. 1998; Tuler et al. 1989; Yanai et al. 2002). Primary neurons do not maintain their mitotic ability in culture and differentiate in a heterogeneous fashion; in contrast, PC12 cells enable detection of adverse effects on the cell cycle, an important target for CPF and other organophosphates (Slotkin 1999, 2004, 2005). With the introduction of the neurotrophin nerve growth factor (NGF), PC12 cells gradually exit the mitotic cycle and differentiate into cells with distinct neuronal phenotypes, possessing axonal projections, electrical excitability, cholinesterase and cholinergic receptors (Fujita et al. 1989; Song et al. 1998; Teng and Greene 1994). Accordingly, the PC12 model is suitable for examination of mechanisms underlying adverse effects on multiple developmental stages ranging from cell replication through end-stage neural differentiation. In the present study, we examined the effects of CPF with and without each of the ameliorating treatments on both undifferentiated and differentiating PC12 cells with regard to DNA synthesis, indices of cell number and size, and AC signaling. Because each neural cell contains a single nucleus, we measured DNA content to evaluate the number of cells (Winick and Noble 1965) and the protein/DNA ratio as an index of cell size (Abreu-Villaça et al. 2005; Jameson et al. 2006a; Slotkin et al. 2007; Song et al. 1998). For AC measurements, we evaluated basal enzymatic activity, the response to global stimulation of G-proteins by fluoride, and maximal enzymatic activity elicited by forskolin, which acts directly on AC, bypassing the need for activation of neurotransmitter receptors or G-proteins (Seamon and Daly 1986).

## Material and Methods

### Cell cultures

Because of the clonal instability of the PC12 cell line (Fujita et al. 1989), we performed the experiments using cells that had undergone fewer than five passages, and all studies were repeated several times with different batches of cells. As described previously (Crumpton et al. 2000a; Qiao et al. 2003; Song et al. 1998), 3 × 10^6^ PC12 cells (1721-CRL; American Type Culture Collection; Manassas, VA) were seeded onto 100-mm poly-d-lysine–coated plates in RPMI-1640 medium (Invitrogen, Carlsbad, CA) supplemented with 10% inactivated horse serum (Sigma Chemical Co., St. Louis, MO), 5% fetal bovine serum (Sigma Chemical Co.), and 50 μg/mL penicillin/streptomycin (Invitrogen). Cells were incubated with 7.5% CO_2_ at 37°C and the medium was changed every 2 days. For studies in the undifferentiated state, the medium was changed 24 hr after seeding to include the various test substances: CPF (Chem Service, West Chester, PA), atropine plus mecamylamine, nicotine, vitamin E, or theophylline (all from Sigma Chemical Co.). Because of poor water solubility, CPF was dissolved in dimethyl sulfoxide (Sigma Chemical Co.) and vitamin E was dissolved in 95% ethanol, achieving final respective concentrations of 0.1% and 0.05%, respectively, in the culture medium; accordingly, all control cultures also included the appropriate vehicles, which had no effect on the PC12 cells (Qiao et al. 2001, 2003; Song et al. 1998). For studies in differentiating cells, 3 × 10^6^ cells were seeded, and 24 hr later, the medium was changed to include 50 ng/mL 2.5 S murine NGF (Invitrogen); each culture was examined under a microscope to verify the subsequent outgrowth of neurites. The test agents were added concurrently with the start of NGF treatment, and cultures were maintained for 6 days, with the indicated agents included with every medium change.

We chose the concentrations of test agents on the basis of previous work. Given our main objective of trying to prevent the adverse effects of CPF, our strategy was to elicit a robust response for each of the effects to be evaluated. Accordingly, we used 30 or 50 μM CPF, concentrations high on the dose–response curves for oxidative stress, inhibition of DNA synthesis, and interference with cell acquisition, but below the threshold for outright cytotoxicity (Bagchi et al. 1995; Crumpton et al. 2000b; Das and Barone 1999; Jameson et al. 2006b; Qiao et al. 2001, 2003, 2005; Slotkin et al. 2007; Song et al. 1998). Atropine and mecamylamine were used at a concentration of 10 μM each, substantially higher than required for blockade of muscarinic and nicotinic acetylcholine receptors, respectively, but at levels devoid of direct, adverse effects on PC12 cells (Song et al. 1998). Nicotine was similarly tested at 10 μM, a concentration that provides partial protection of PC12 cells against oxidative stress and antimitotic effects of CPF (Qiao et al. 2003, 2005). The antioxidant vitamin E was evaluated at concentrations of 10 and 30 μM, as established for demonstrable prevention of oxidative stress in earlier work (Qiao et al. 2005). The phosphodiesterase inhibitor theophylline was tested at both 1 and 10 mM, concentrations previously found to affect the differentiation of MDA-MB-231 cells, which, like PC12 cells, use cyclic AMP as a differentiation signal (Slotkin and Seidler 2000; Slotkin et al. 2000).

### DNA synthesis

To initiate the measurement of DNA synthesis, the medium was changed to include 1 μCi/mL [^3^H]thymidine (specific activity, 2 Ci/mmol; GE Healthcare, Piscataway, NJ) along with the continued inclusion of the test substances. After 1 hr, the medium was aspirated and cells were harvested in ice-cold water. Duplicate aliquots of each sample were treated with 10% trichloroacetic acid and sedimented at 1,000 × *g* for 15 min to precipitate macromolecules. The resulting pellet was washed once with additional trichloro-acetic acid and then with 75% ethanol. The final pellet was hydrolyzed with 1 M potassium hydroxide overnight at 37°C and neutralized with 6 M hydrochloric acid, and the DNA was precipitated with ice-cold 5% trichloroacetic acid and resedimented. The supernatant solution, comprising solubilized RNA and protein, was discarded. The DNA-containing pellet was hydrolyzed in 5% trichloroacetic acid for 15 min at 90°C and resedimented, and an aliquot of the supernatant solution was counted for radiolabel. Another aliquot was assayed for DNA spectrophotometrically by absorbance at 260 nm. Previous work has demonstrated quantitative recovery of DNA by these techniques (Bell et al. 1986; Slotkin et al. 1984). Incorporation values were corrected to the amount of DNA present in each culture to provide an index of DNA synthesis per cell (Winick and Noble 1965).

### DNA and protein content

For determinations of DNA content and the protein/DNA ratio, the medium was aspirated and the culture was rinsed with a buffer consisting of 154 mM sodium chloride and 10 mM sodium phosphate (pH 7.4). Cells were harvested in ice-cold buffer and homogenized (Polytron, Brinkmann Instruments, Westbury, NY), and aliquots were withdrawn for measurements of DNA and protein using dye-binding methods (Smith et al. 1985; Trauth et al. 2000).

### Adenylyl cyclase activity

The AC assay procedures and stimulant concentration profiles have been described in detail previously (Auman et al. 2000; Meyer et al. 2005; Zeiders et al. 1999). To prepare the cell membrane fraction, the homogenates were sedimented at 40,000 × *g* for 10 min and the pellet was washed and resedimented. The membrane pellets were resuspended using a smooth glass homogenizer fitted with a Teflon pestle, in a buffer consisting of 250 mM sucrose, 2 mM magnesium chloride, and 50 mM Tris (pH 7.5). Incubations contained 50 μg of membrane protein (Smith et al. 1985) in a medium consisting of 40 mM Tris HCl (pH 7.4), 10 mM theophylline, 1 mM adenosine 5′-triphosphate, 10 μM guanosine 5′-triphosphate, 2 mM MgCl_2_, 1 mg/mL bovine serum albumin, and a creatine phosphokinase-ATP-regenerating system consisting of 10 mM sodium phosphocreatine and 8 IU/mL phosphocreatine kinase (all from Sigma Chemical Co.), in a total volume of 250 μL. The enzymatic reaction was stopped by placing the samples in a 90–100°C water bath for 5 min, followed by sedimentation at 3,000 × *g* for 15 min, and the supernatant solution was assayed for cyclic AMP by radioimmunoassay (GE Healthcare). AC activity was evaluated under three different conditions: basal activity, the response to 10 mM NaF, and the response to 100 μM forskolin (Sigma Chemical Co.).

### Data analysis

Results are reported as means and SEs. Because of the multiple treatments in each experiment, significant differences were first established by a global analysis of variance (ANOVA) incorporating all treatments, followed by Fisher’s protected least-significant difference to evaluate differences between specific treatment groups. Significance was assumed at the level of *p* < 0.05. To facilitate visual comparisons among the different studies, all experiments were normalized so as to share a standard set of control values, which were calculated as the average across all the experiments. The actual values varied by as much as 50% among different platings of cells. However, all statistical comparisons were conducted on the original data.

## Results

### Atropine plus mecamylamine

**Figure 1:**
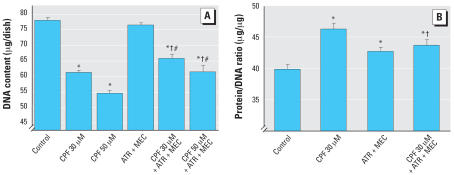
Effects of CPF (30 or 50 μM) with and without cotreatment with 30 μM atropine (ATR) plus 30 μM mecamylamine (MEC) on DNA content [*A*; ANOVA: treatment, *p* < 0.0001 (*n* = 12–24)] and total protein/DNA ratio [*B*; ANOVA: treatment, *p* < 0.0001 (*n* = 11–12)] in differentiating PC12 cells. Cells were treated with the indicated agents along with NGF for a total of 6 days. *Significantly different from controls. ^†^Cotreated values significantly different from CPF alone. ^#^Cotreated values significantly different from ATR plus MEC.

In earlier work, we showed that the combination of atropine plus mecamylamine failed to prevent the decline in DNA synthesis evoked by CPF exposure in undifferentiated PC12 cells (Song et al. 1998). In differentiating cells, exposure to 30 or 50 μM CPF for 6 days elicited a robust, dose-dependent decrement in cell number as assessed by DNA content (Figure 1A). Although the combination of atropine plus mecamylamine had no effect by itself, it provided significant but partial protection from the adverse effects of CPF, reducing the impact of the organophosphate by about 40%. Accompanying the decrease in cell number, CPF exposure caused a significant increase in the protein/DNA ratio (Figure 1B). For this measure, the cholinergic antagonists by themselves also produced a partial effect, increasing the ratio by about one-third of the effect seen with CPF. Nevertheless, the atropine plus mecamylamine combination still provided significant protection against the CPF-induced increase, reducing the value to that seen with the antagonists alone.

**Figure 2:**
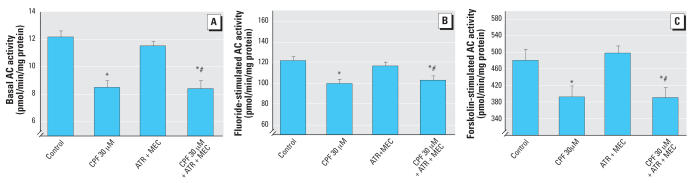
Effects of 30 μM CPF with and without cotreatment with 30 μM atropine (ATR) plus 30 μM mecamylamine (MEC) on basal AC activity [*A*; ANOVA: treatment, *p* < 0.0001 (*n* = 9–11)], fluoride-stimulated AC activity [*B*; ANOVA: treatment, *p* < 0.0003 (*n* = 9–11)], and forskolin-stimulated AC activity [*C*; ANOVA: treatment, *p* < 0.004 (*n* = 10–11)] in differentiating PC12 cells. Cells were treated with the indicated agents along with NGF for a total of 6 days. *Significantly different from controls. #Cotreated values significantly different from ATR plus MEC .

CPF exposure also impaired AC signaling in differentiating PC12 cells. For basal AC activity, 30 μM CPF reduced activity by about one-third (Figure 2A); the combination of atropine plus mecamylamine had no effect by itself, but in this case, failed to protect the cells from the adverse actions of CPF. The effect of CPF on fluoride-stimulated AC activity was notably less than for basal activity (20% decrease), but the same pattern was seen for the cholinergic blocking agents: there was no effect by themselves and no prevention of the reduction caused by CPF (Figure 2B). For forskolin-stimulated AC activity, CPF similarly produced deficits that were not blocked by atropine plus mecamylamine (Figure 2C).

### Nicotine

**Figure 3:**
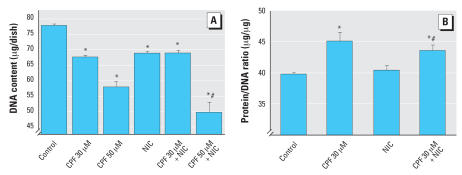
Effects of 30 μM or 50 μM CPF with and without cotreatment with 10 μM nicotine (NIC) on DNA content [*A*; ANOVA: treatment, *p* < 0.0001 (*n* = 12–30)] and total protein/DNA ratio [*B*; ANOVA: treatment, *p* < 0.0002 (*n* = 16–18)] in differentiating PC12 cells. Cells were treated with the indicated agents along with NGF for a total of 6 days. *Significantly different from controls. ^#^Cotreated values significantly different from NIC alone.

**Figure 4:**
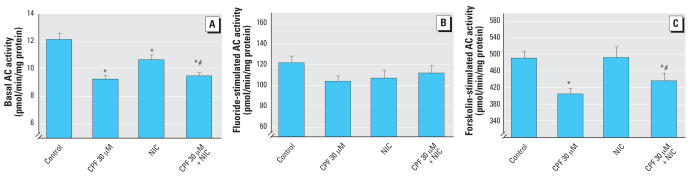
Effects of 30 μM CPF with and without cotreatment with 10 μM nicotine (NIC) on basal AC activity [*A*; ANOVA: treatment, *p* < 0.0001 (*n* = 10–12)] , fluoride-stimulated AC activity [*B*; ANOVA: treatment, not significant (*n* = 11–12)], and forskolin-stimulated AC activity [*C*; ANOVA: treatment, *p* < 0.004 (*n* = 10–12)] in differentiating PC12 cells. Cells were treated with the indicated agents along with NGF for a total of 6 days. *Significantly different from controls. #Cotreated values significantly different from NIC alone.

We previously showed partial protection by nicotine against the antimitotic effects of CPF in undifferentiated PC12 cells, despite the fact that nicotine, by itself, had a slight inhibitory effect (Qiao et al. 2003). In differentiating PC12 cells, nicotine caused a significant deficit in DNA content after 6 days of exposure (Figure 3A). When nicotine was coadministered with the lower concentration of CPF (30 μM), the net outcome was similar to the deficits seen with either treatment alone, rather than showing the expected additive effect; this could imply some protection. However, with the higher CPF exposure (50 μM), there was a clear-cut worsening of the outcome, with greater deficits in cell number than were seen with either CPF or nicotine given separately. For the protein/DNA ratio, nicotine by itself had no significant effect, nor did it prevent the increase evoked by CPF (Figure 3B). Nicotine also produced a significant decrement in basal AC activity, but to a lesser extent than for CPF (Figure 4A). Nicotine did not prevent the reduction caused by CPF, but the combined effect was less than would have been expected from additive actions of the two individual agents. For fluoride-stimulated AC activity (Figure 4B) or forskolin-stimulated activity (Figure 4C), nicotine did not demonstrate any significant reversal of the effects of CPF.

### Vitamin E

**Figure 5:**
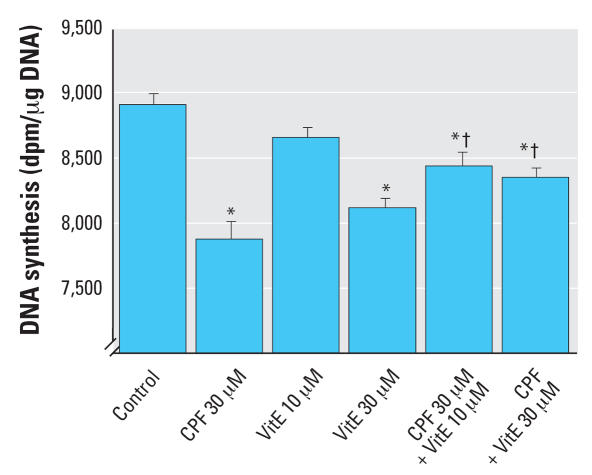
Effects of 30 μM CPF with and without cotreatment with 10 or 30 μM vitamin E (VitE) on DNA synthesis in undifferentiated PC12 cells. ANOVA: treatment, *p* < 0.0001 (*n* = 5–16). Cells were treated with the indicated agents for 24 hr, and then [^3^H]thymidine incorporation into DNA was measured for 1 hr. *Significantly different from controls. ^†^Cotreated values significantly different from CPF alone.

**Figure 6:**
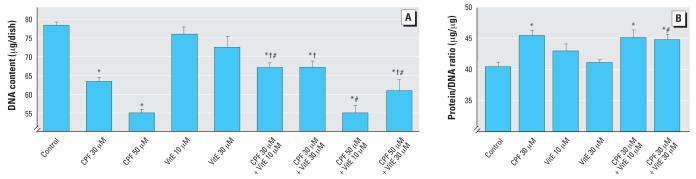
Effects of 30 or 50 μM CPF with and without cotreatment with 10 or 30 μM vitamin E (VitE) on DNA content [*A*; ANOVA: treatment, *p* < 0.0001 (*n* = 8–16)] and total protein/DNA ratio [*B*; ANOVA: treatment, *p* < 0.0004 (*n* = 8)] in differentiating PC12 cells. Cells were treated with the indicated agents along with NGF for a total of 6 days. *Significantly different from controls. ^†^Cotreated values significantly different from CPF alone. ^#^Cotreated values significantly different from VitE alone.

Because we had not previously evaluated the effects of vitamin E on DNA synthesis in undifferentiated PC12 cells, we compared its effects alone or in combination with CPF (Figure 5). At a concentration of 30 μM, CPF evoked significant inhibition of DNA synthesis, in keeping with earlier findings (Qiao et al. 2001, 2003; Slotkin et al. 2007; Song et al. 1998). By itself, vitamin E also produced a dose-dependent decrement, with an effect nearly equivalent to that of CPF at the same concentration. Nevertheless, vitamin E protected the cells from the adverse effects of CPF. At either 10 or 30 μM vitamin E, the inhibition caused by CPF was blunted, resulting in a net value not distinguishable from the effect of vitamin E alone but significantly different from that of CPF alone. When vitamin E was added during cell differentiation, we saw a trend toward decreased DNA content, but the effect was too small to achieve statistical significance (Figure 6A). Again, this anti-oxidant provided significant protection from the adverse effects of CPF. At the lower concentration (30 μM) of CPF, addition of either 10 or 30 μM vitamin E produced a net value midway between that seen with either vitamin E or CPF alone, so that the effects of CPF were significantly reduced. At the higher CPF concentration (50 μM), 10 μM vitamin E was ineffective but 30 μM vitamin E still produced significant protection. We also evaluated the protein/DNA ratio for the effects of 30 μM CPF with and without vitamin E (Figure 6B); for this parameter, vitamin E did not produce any discernible protection from the increase caused by CPF.

**Figure 7:**
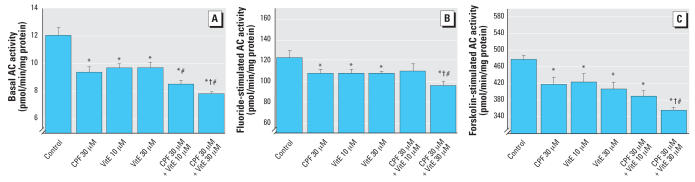
Effects of 30 μM CPF with and without cotreatment with 10 or 30 μM vitamin E (VitE) on basal AC activity [*A*; ANOVA: treatment, *p* < 0.0001 (*n* = 7–8)], fluoride-stimulated AC activity [*B*; ANOVA: treatment, *p* < 0.01 (*n* = 6–8)], and forskolin-stimulated AC activity [*C*; ANOVA: treatment, *p* < 0.0001 (*n* = 7–9)] in differentiating PC12 cells. Cells were treated with the indicated agents along with NGF for a total of 6 days. *Significantly different from controls. ^†^Cotreated values significantly different from CPF alone. ^#^Cotreated values significantly different from VitE alone.

Surprisingly, vitamin E elicited deficits in basal AC activity quite similar to those seen with CPF (Figure 7A). Here the combination of CPF plus vitamin E resulted in even greater loss of activity, so that rather than protecting the cells from the adverse effects of CPF, the actions were additive. A similar pattern was seen for fluoride-stimulated AC activity (Figure 7B) as well as the response to forskolin (Figure 7C). In each case, vitamin E produced inhibition similar to that seen with CPF and either failed to prevent the effect of CPF (fluoride) or worsened the net effect (forskolin).

### Theophylline

**Figure 8:**
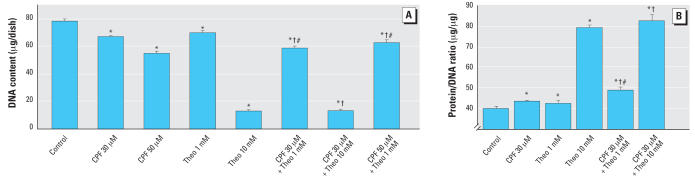
Effects of 30 or 50 μM CPF with and without cotreatment with 1 or 10 mM theophylline (Theo) on DNA content [*A*; ANOVA: treatment, *p* < 0.0001 (*n* = 11–22)] and total protein/DNA ratio [*B*; ANOVA: treatment, *p* < 0.0001 (*n* = 11–12)] in differentiating PC12 cells. Cells were treated with the indicated agents along with NGF for a total of 6 days. Note the difference in scale from the other figures. *Significantly different from controls. †Cotreated values significantly different from CPF alone. ^#^Cotreated values significantly different from Theo alone.

For theophylline, we also evaluated the effects on DNA synthesis in undifferentiated PC12 cells. By itself, this agent caused massive reductions in the incorporation of [^3^H]thymidine into DNA: 8,863 ± 278 dpm/μg DNA for controls (*n* = 8), 3,638 ± 133 for 1 mM theophylline (*p* < 0.0001, *n* = 8), and 753 ± 43 for 10 mM theophylline (*p* < 0.0001, *n* = 8). Accordingly, we were unable to detect any potential ability of theophylline to prevent the much smaller decrement in DNA synthesis caused by CPF, but this likely represents the technical limitation imposed by the large effect of theophylline by itself. In differentiating cells, theophylline also had a significant adverse impact on cell number as monitored by DNA content (Figure 8A). The lower concentration (1 mM) produced a deficit similar to that caused by 30 μM CPF alone and raising the theophylline concentration to 10 mM resulted in a decline of almost 90%. The combination of 30 μM CPF with 1 mM theophylline produced an additive deficit in cell number, so that the DNA content was significantly lower than with either agent by itself. However, when the CPF concentration was raised to 50 μM, theophylline provided significant protection, so that the net value was midway between that seen with theophylline alone or CPF alone.

**Figure 9:**
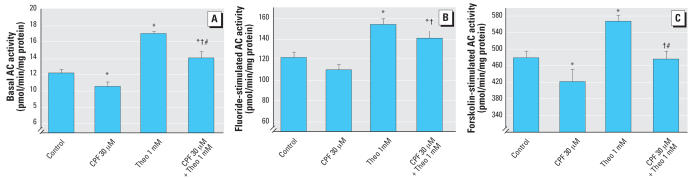
Effects of 30 μM CPF with and without cotreatment with 1 mM theophylline (Theo) on basal AC activity [*A*; ANOVA: treatment, *p* < 0.0001 (*n* = 8–10)], fluoride-stimulated AC activity [*B*; ANOVA: treatment, *p* < 0.0001 (*n* = 8–10)], and forskolin-stimulated AC activity [*C*; ANOVA: treatment, *p* < 0.0001 (*n* = 9–10)] in differentiating PC12 cells. Cells were treated with the indicated agents along with NGF for a total of 6 days. *Significantly different from controls. †Cotreated values significantly different from CPF alone. ^#^Cotreated values significantly different from Theo alone.

Equally dramatic effects of theophylline were seen for measures of AC activity. By itself, 1 mM theophylline evoked significant up-regulation of basal AC activity, so that the addition of this agent reversed the inhibitory effects of CPF completely (Figure 9A). Essentially the same reversal was obtained for fluoride-stimulated AC activity (Figure 9B) and for the forskolin response (Figure 9C). In each case, theophylline produced a significant elevation that completely offset the effect of CPF, even to the extent of producing a net increase in activity from the combined exposure for the basal and fluoride measurements.

## Discussion

Our findings support the concept that multiple mechanisms contribute to the net adverse effects of CPF on neural cell development both in the undifferentiated state and during differentiation into neuronal phenotypes. In earlier studies with undifferentiated PC12 cells (Song et al. 1998), we found that blockade of acetylcholine receptors with atropine plus mecamylamine failed to prevent the decline in mitotic index elicited by CPF exposure, indicating essentially no contribution of cholinesterase inhibition and cholinergic hyperstimulation to this end point. In contrast, in the present study, when we introduced NGF to initiate differentiation, the cholinergic antagonists provided partial protection against the deficits in cell numbers, as evidenced by a reduced effect of CPF on DNA content. Thus, there is a transition in contributory mechanisms to the adverse effects of CPF, with relatively little reliance on cholinergic hyperstimulation in the undifferentiated state, but there is a significant role for this component once neural cell differentiation gets under way. Our findings are consonant with the increased role of cholinergic factors in the effects of CPF on later versus earlier stages of brain development *in vivo* (Whitney et al. 1995) and are further reinforced by the results for nicotine, which stimulates nicotinic acetylcholine receptors directly. In undifferentiated PC12 cells, nicotine has only a small effect on DNA synthesis, but with the initiation of differentiation this effect becomes much larger (Qiao et al. 2003). Furthermore, rather than producing additive effects on DNA synthesis, nicotine actually protects undifferentiated cells from the adverse effects of CPF (Qiao et al. 2003), indicating that the antimitotic actions depend on other mechanisms. In the present study, we tried the same strategy in differentiating PC12 cells and instead found a mixed effect, just as would be expected from additive cholinergic hyper-stimulation from the two agents, superimposed on noncholinergic, protective actions of nicotine (e.g., antioxidant properties). The combination of 30 μM CPF plus 10 μM nicotine produced a decrement in DNA content indistinguishable from that achieved by either agent alone, but clearly less than would be anticipated from additive deficits. However, raising the CPF concentration to 50 μM resulted in the expected cumulative effects.

The obvious issue, then, is to identify the noncholinergic mechanism or mechanisms that are superimposed on the cholinergic components. Nicotine possesses both prooxidant and antioxidant properties that depend both on its concentration and on the underlying cellular oxidative status (Gitto et al. 2002; Guan et al. 2003; Qiao et al. 2005; Xie et al. 2005). Thus, although nicotine itself can produce oxidative stress in otherwise untreated PC12 cells (Abreu-Villaça et al. 2005), it limits the ability of CPF to produce even greater stress (Qiao et al. 2005). We therefore reasoned that, if the mixed effect of nicotine reflected the combination of antioxidant actions superimposed on cholinergic hyper-stimulation, then a pure antioxidant such as vitamin E might demonstrate clear protection. Indeed, in undifferentiated cells, where the cholinergic contribution is low, vitamin E produced substantial protection against the anti-mitotic effects of CPF. In differentiating cells, there was similarly a clear-cut beneficial effect in preventing the decline in DNA content, with progressively more vitamin E required as the CPF concentration was raised. These results demonstrate conclusively that oxidative stress plays a pivotal role in the noncholinergic component of CPF’s interference with cell cycle and maintenance of appropriate numbers of neural cells. Nevertheless, there were potential problems in pursuing a strategy relying solely on preventive effects of vitamin E. At the higher concentration of 30 μM, vitamin E by itself caused significant reductions in mitotic index in undifferentiated cells and a tendency toward reduced DNA content in differentiating cells. Intracellular oxidative status increases substantially during the transition from cell replication to differentiation (Abreu-Villaça et al. 2005; Qiao et al. 2005) and naturally occurring oxidative stress may actually be involved in many of the cellular events associated with these events (Katoh et al. 1997). Thus, as with nicotine, the actions of anti-oxidants such as vitamin E may be dual in nature, with protective actions against CPF but some adverse effects by themselves.

Cyclic AMP provides one of the major endogenous signals for the switch from neural cell replication to differentiation (Mark and Storm 1997; McManus et al. 1999; Stachowiak et al. 2003), and CPF targets G-protein coupled receptors, G-proteins, and adenylyl cyclase as major noncholinergic components of its effects on brain development (Gupta 2004; Slotkin 1999, 2004, 2005; Yanai et al. 2002). Accordingly, it is not surprising that we found gross inhibition of DNA synthesis in undifferentiated PC12 cells upon addition of theophylline, a phosphodiesterase inhibitor that enhances the intracellular cyclic AMP concentration. In differentiating cells, however, we found a mixed effect as would be anticipated from a combination of the direct actions of theophylline but protection against the adverse effects of CPF. With 30 μM CPF plus 1 mM theophylline, the net reduction in DNA content was roughly equivalent to the additive effects of the two agents. However, at the higher CPF concentration (50 μM), theophylline provided clear but partial protection, reducing the deficits to midway between the two separate treatments, demonstrably less than would have been anticipated from additive effects. The net result thus resembles that seen with nicotine, where the direct effect of the therapeutic intervention has some adverse actions but nevertheless protects from further injury by CPF. In contrast, though, at the higher theophylline concentration (10 mM), the adverse effects of theophylline itself were so large as to obscure any potential benefit when combined with CPF.

Although CPF has robust effects on cell replication and cell number, it does not reduce cell growth until much higher concentrations than those in the current study are used (Song et al. 1998). In differentiating PC12 cells, cell enlargement occurs in part because of the generation of neuritic projections, an essential part of the transition to the neuronal phenotype (Teng and Greene 1994). Although CPF interferes with axonogenesis, it can promote formation of shorter neuritic projections so that the effects on neurite formation are mixed at concentrations such as those used here (Das and Barone 1999; Howard et al. 2005; Song et al. 1998). Accordingly, although CPF caused a substantial reduction in DNA content, the protein/DNA ratio was increased by CPF exposure; this increase was to a lesser extent than the reduction in DNA content, implying that there are indeed direct effects of CPF on the protein components over and above the impairment of cell acquisition. The combination of atropine plus mecamylamine also produced an increase in the ratio, albeit smaller than that caused by CPF. Importantly, though, the blocking agents produced a partial reversal of the increase in protein/DNA ratio elicited by CPF, bringing the value down to that seen with just atropine plus mecamylamine. However, for the other agents, there was a different outcome. Neither nicotine nor vitamin E by themselves had any effect (unlike their actions on DNA synthesis and content) and they also failed to provide any protection against CPF. Theophylline evoked an increase in the ratio, but when combined with CPF the effects were additive. Thus, only one of the strategies that were successful in providing partial protection against CPF for DNA synthesis and content worked for the effects on this index of cell growth and neurite formation.

The third target we examined was AC activity. Consistent with its effects in the developing brain (Slotkin 1999, 2004, 2005; Song et al. 1997; Yanai et al. 2002), CPF elicited deficits in AC signaling in differentiating PC12 cells. Here, though, neither the cholinergic antagonists nor nicotine proved beneficial in protecting the cells from CPF. Vitamin E had deleterious actions, and when used in combination with CPF, the deficits were additive. The only promising result was obtained with theophylline, and that was because it evoked a substantial increase in AC by itself. When superimposed on the adverse effects of CPF, the stimulation evoked by theophylline thus restored AC activity to normal or supranormal values. This is likely to play an important role in neuroprotection against organophosphates: forskolin, which stimulates adenylyl cyclase directly, preserves neural cells from the adverse effects of diisopropylfluorophosphate, potentially by promotion of differentiation, which up-regulates acetylcholinesterase itself (Curtin et al. 2006). In addition, we found that CPF affected the response to fluoride significantly less than it did basal AC activity. Fluoride maximally activates both stimulatory (G_s_) and inhibitory (G_i_) G-proteins; this result implies that the organophosphate treatment produces a shift in the relative expression and/or function of G_s_ and G_i_, which would likely influence the activities of receptors that stimulate or inhibit AC. Although we did not pursue this issue in the present study, it is clearly an important consideration for future work, both in terms of additional mechanisms for CPF-induced developmental neurotoxicity and for contributions to subsequent neurotransmitter mechanisms underlying behavioral anomalies.

In the developing brain, the neurotoxicity of CPF undergoes distinct transitions in mechanisms and cellular targets, so that although adverse effects are elicited at virtually any maturational stage, the regions, neurotransmitters, and behaviors that are affected shift according to the period in which exposure occurs (Slotkin 1999, 2004, 2005). Here, with the PC12 model, we were able to identify the critical stages in which some of these diverse mechanisms operate and consequently to evaluate specific amelioration strategies that might provide protection against the adverse effects of CPF. Importantly, each of the mechanisms made a partial contribution to the net outcome for either neural cell replication or differentiation. Cholinergic antagonists, although totally ineffective in preventing the antimitotic effects of CPF in undifferentiated cells (Song et al. 1998), were successful once differentiation was under way, in partially reversing the deficits in cell number and the rise in protein/DNA ratio; this is entirely consistent with the transition to a cholinergic phenotype in PC12 cells (Jameson et al. 2006a, 2006b; Teng and Greene 1994) and with the increasing role of cholinergic mechanisms in the adverse effects of CPF *in vivo* as brain development progresses (Slotkin 1999, 2004, 2005; Whitney et al. 1995). Nicotine was partially effective in protecting cell replication in undifferentiated cells but likely through antioxidant actions (Qiao et al. 2005), so that a similar effect was found with vitamin E. With increasing differentiation, the effects of nicotine acting as a cholinergic agonist offset some of the positive consequences of its antioxidant actions, so that the remaining beneficial effect depended on whether the CPF concentration was low or high. For nicotine, too, any potential value for amelioration of organophosphate-induced neurodevelopmental injury must be tempered by the fact that nicotine itself is a neuroteratogen (Slotkin 1999, 2004); this will limit its utility to short-term use. Alternatively, further mechanistic investigations may enable substitution of analogs that preserve the protectant effects while reducing the direct, adverse impact on neurodevelopment. Nicotine is also generally neuroprotectant in the intact brain by causing the release of neurotrophic factors (Belluardo et al. 2000), an effect that we would not likely detect in a monoculture of PC12 cells; this implies that, used properly, nicotine or nicotine analogs may be even more effective *in vivo* than seen here. Similar to nicotine, vitamin E had complex effects after addition of NGF because, although it protected the cells from oxidative stress caused by CPF, it also interferes with the role of endogenous oxidative factors in neural cell differentiation. Finally, theophylline was the only agent that was able to fully restore the impairment of AC activity caused by CPF, but it did so only because it elevated AC by itself, not because it interfered directly with the effect of CPF. Here again, although theophylline by itself had adverse effects on DNA synthesis and indices of cell number, the possibility remains that analogs may prove better than the parent compound. Theophylline is relatively nonspecific as a phosphodiesterase inhibitor, and we recently found that CPF targets only a few phosphodiesterase and AC subtypes (Slotkin and Seidler 2007). Thus, it may be possible to find an alternative that maintains the protectant effect against CPF while avoiding the direct adverse effect of theophylline itself.

Although *in vitro* models are not a substitute for animal studies of protection against organophosphate-induced developmental neurotoxicity, our results provide proof that the proposed divers mechanisms by which CPF damages developing neurons are indeed exerted directly by this agent and not indirectly through complex effects on the maternal–fetal unit, and further, provide a “proof of principle” that interventions aimed at these mechanisms may indeed prove valuable for amelioration of the adverse effects. However, the importance of our findings transcends these issues. Organophosphates represent 50% of all insecticides used worldwide (Casida and Quistad 2004), and there is increasing likelihood of human exposures to organophosphate nerve agents such as those used in the terrorist incidents in Matsumoto and the Tokyo subway in Japan; therefore, identifying potential ameliorating therapies is critical. Our findings suggest that a cocktail of agents targeting the various mechanisms for neuronal injury by organophosphates may prove successful. Nevertheless, devising an appropriate combination that avoids harmful effects of the ameliorating agents themselves, establishing the proper doses and pharmacokinetic profiles, and evaluating whether the treatment cocktail works equally for different organosphosphates or for the different exposure scenarios in pesticide use versus a terrorist/nerve gas incident will likely be a daunting task.
